# Effects of storage time and temperature on coagulation tests and factors in fresh plasma

**DOI:** 10.1038/srep03868

**Published:** 2014-01-27

**Authors:** Limin Feng, Ying Zhao, Hongcan Zhao, Zhexin Shao

**Affiliations:** 1 Department of Laboratory Medicine, The First Affiliated Hospital, College of Medicine, Zhejiang University, Hangzhou 310003, China; 2 Department of Laboratory Medicine, The First People's Hospital of Hangzhou, Hangzhou 310003, China; 3 Department of Hospital Office, The First Affiliated Hospital, College of Medicine, Zhejiang University, Hangzhou 310003, China

## Abstract

Coagulation tests and factors measurements have been widely applied in clinical practice. Pre-analytical conditions are very important in laboratory assessment.Here,we aim to determine the effects of storage time and temperature on activated partial thromboplastin time (APTT), fibrinogen (Fbg), prothrombin time (PT), the international normalized ratio (INR), thrombin time (TT), factor VIII activity (FVIII:C), and factor IX activity (FIX:C) in fresh plasma. Seventy-two blood samples were tested after storage for 0 (baseline), 2, 4, 6, 8, 12, and 24 h at 25°C (room temperature) and 4°C (refrigeration) in two centers. The mean percentage change of greater than 10% and the numbers of samples with greater than 10% percentage changes more than 25% were used to determine clinically relevant difference. We demonstrated that samples for Fbg, PT/INR, and TT could be safely stored for ≤24 h; FVIII:C for ≤2 h; FIX:C for ≤4 h both at 4°C and 25°C; and APTT for ≤12 h at 4°C and ≤8 h at 25°C.

Pre-analytical conditions are very important in laboratory assessment of hemostatic and coagulation systems^1^. Pre-analytical variables including specimen collection, anticoagulant type and concentration, hematocrit, filling status of the sampling tube, transportation, centrifugation, as well as storage and assay method can all affect coagulation test and factor analysis results^1^,^2^. Activated partial thromboplastin time (APTT), fibrinogen (Fbg), prothrombin time (PT), international normalized ratio (INR, transformed by PT), and thrombin time (TT) measurements are routine coagulation tests used to assess pathological alterations of hemostatic and coagulation systems to guide clinical therapy^2^. In addition, the PT/INR ratio is used to monitor oral anticoagulant therapy for reducing the risk of thromboembolic events and minimizing the incidence of bleeding complications^3^. Coagulation factor VIII (FVIII) and factor IX (FIX) play a major role in the endogenous and exogenous thrombin pathways, are used to diagnose hemophilia, are often associated with chronic liver disease, act as risk factors for thrombosis, and are used as quality markers of fresh-frozen plasma (FFP) and cryoprecipitate^1^,^4^,^5^,^6^. After blood is collected, factor VIII activity (FVIII:C) and factor IX activity (FIX:C) are gradually reduced; thus, different storage temperatures and durations affect coagulation test results^6^,^7^,^8^. For these pre-analytical variables, the Clinical and Laboratory Standards Institute (CLSI) H21-A5 has recommended that specimens should be analyzed within 24 h for PT and 4 h for APTT and other assays if stored at room temperature (25°C). However, they have not recommended a storage time for refrigerated storage (2–8°C)^9^.

Many studies have suggested acceptable storage temperatures and times for routine coagulation tests^8^,^10^,^11^. In addition, although the influences of storage time and temperature on FVIII:C and FIX:C in FFP have been reported^6^, the stability of factor activities in fresh plasma without processing or the addition of stabilizer has not been evaluated systematically. Numerous patients with hematological diseases and liver diseases are admitted to one of two comprehensive hospitals located in Hangzhou, China. Therefore, timely and accurate coagulation tests and factor detection in fresh plasma samples are very important to diagnose and treat hemophilia and to monitor oral anticoagulant therapy, chronic liver disease, and thrombotic disease. The large number of specimens received can lead to delays in sample testing in the clinical laboratory. Thus, the aims of this study were to investigate whether storage temperature and time influence the results of routine coagulation tests and factor analysis, and whether any changes caused by delayed analyses result in a clinically relevant difference, as well as to establish our own acceptable storage temperature and time guidelines. In our study, we determined the values of APTT, Fbg, PT/INR, TT, FVIII:C, and FIX:C in samples stored for 0, 2, 4, 6, 8, 12, and 24 h at 25°C and 4°C, respectively. Two laboratories with the same analysis system of a Sysmex CA7000 instrument (Sysmex, Kobe, Japan) and Siemens reagents (Siemens, Marburg, Germany) participated in the study.

## Results

### Between- and within-batch imprecision

Between- and within-batch imprecision of coagulation tests and factors were all consistent with the manufacturer's product information. Within- and between-batch imprecision were <3% and 10%, respectively.

### Stability studies

Table 1 lists the results and statistical differences of the coagulation tests and factor activities of the plasma samples stored for 2, 4, 6, 8, 12, and 24 h after collection at 25°C and 4°C, compared with the baseline results. Table 2 shows the stability of PT/INR, APTT, Fbg, TT, FVIII:C, and FIX:C when the samples were stored under these conditions.

**Figure 1:**
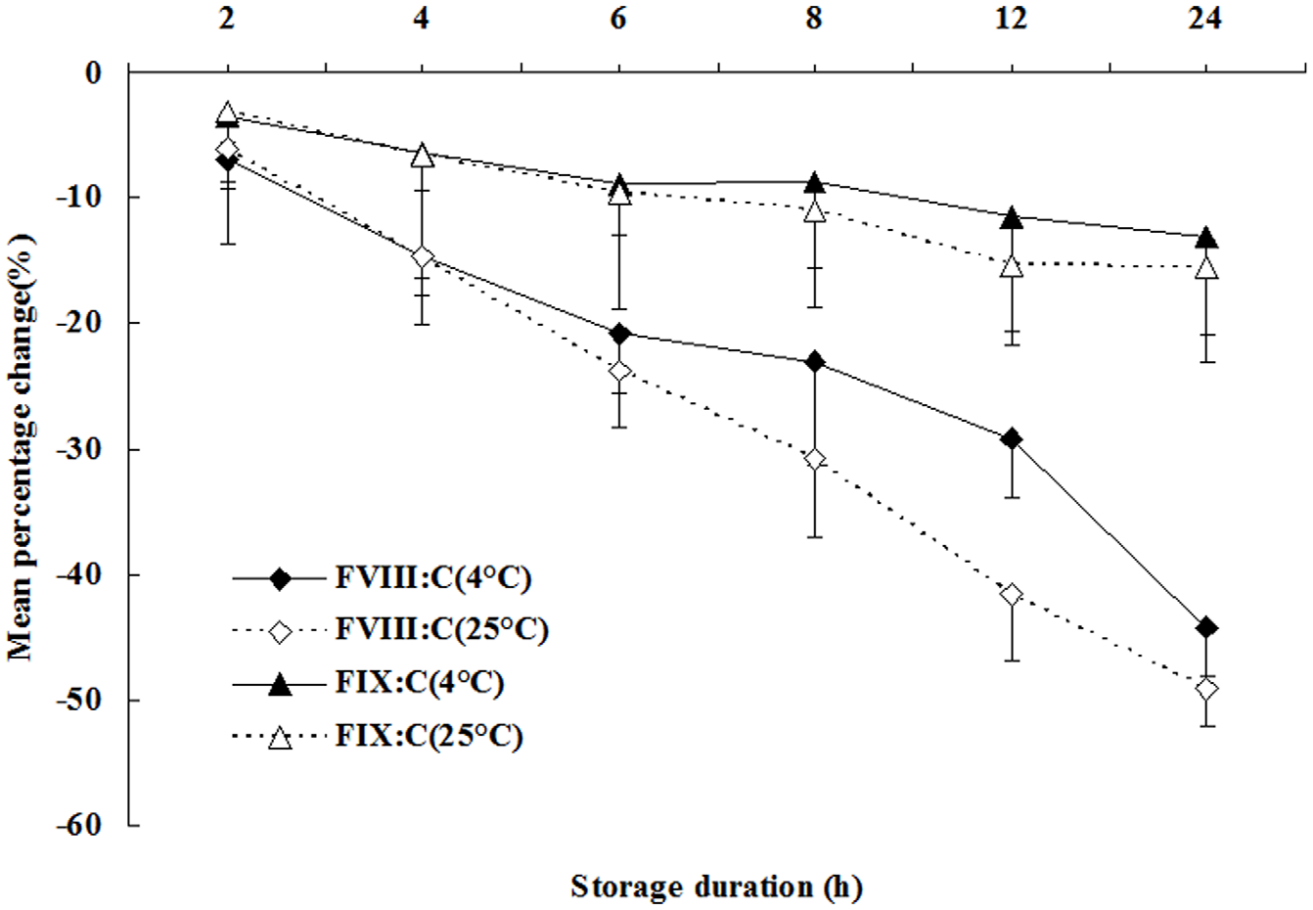
Mean percentage change of FVIII:C and FIX:C for plasma samples stored at 25°C and 4°C for 2, 4, 6, 8, 12, and 24 h.

The mean percentage changes in the values of Fbg, PT/INR, and TT were all less than 10%, and the mean percentage changes in the values of APTT following sample storage for 2, 4, 6, 8, and 12 h at 4°C and 2, 4, 6, and 8 h at 25°C were all less than 10%, compared to the baseline values. However, the mean percentage changes of FVIII:C and FIX:C all exceeded 10%, except FVIII:C storage for 2 h at 25°C and 4°C; FIX:C storage for 2, 4, 6, and 8 h at 4°C; and FIX:C storage for 2, 4, and 6 h at 25°C. Table 2 shows that the numbers of samples with percentage changes greater than 10% for APTT, Fbg, PT/INR, and TT determination were all less than 25% of the samples after storage for 2, 4, 6, 8, 12, and 24 h at 25°C and 4°C, except for APTT determination for samples stored for 12 h at 25°C, and 24 h at 4°C and 25°C. In the cases of FVIII:C and FIX:C, the number of samples with a percentage change greater than 10% for FVIII:C was less than 25% of the samples only after storage for 2 h, and for FIX:C it was after storage for 2 h and 4 h. Furthermore, Figure 1 shows the trend of mean percentage changes of FVIII:C and FIX:C in samples stored for 2, 4, 6, 8, 12, and 24 h at 25°C and 4°C. With extended storage time, at both 25°C and 4°C, FVIII:C and FIX:C were reduced significantly; FVIII:C was even reduced to as low as 49.13% of the baseline value after storage for 24 h at 25°C; meanwhile, FIX:C was reduced to 15.59%.

## Discussion

Coagulation tests and factors measurements have been widely applied in clinical practice; therefore, it is necessary to evaluate the effects of temperature and time from collection on the outcome of these results. Our multicenter study investigated the effects of split tube storage for 2, 4, 6, 8, 12, and 24 h at 25°C and 4°C. Of note, this study was the first to investigate the stability of FVIII:C and FIX:C at 25°C and 4°C in fresh plasma. Although many domestic and foreign scholars have studied the effects of pre-analytical variables on coagulation test analysis, no studies have described the stability of FVIII:C and FIX:C at 25°C and 4°C. A summary of these studies is listed in Table 3^2^,^8^,^10^,^12^,^13^,^15^,^16^,^17^. Nonetheless, there are no unified guidelines for clinically acceptable bias. Some scholars have suggested the use of mean percentage change to evaluate the stability of coagulation tests and factor determination and have considered a mean percentage change of less than 10% to be clinically relevant^12^,^13^,^14^. Moreover, van Geest-Daalderop et al. proposed that if the number of individuals with a greater than 10% percentage change was less than 25% of the total sample number, the effect should be termed moderate and clinically relevant^12^. However, Zürcher^18^ suggested that the imprecision may have a greater impact on the results than the changes in stability studies. In this study, to minimize analytical performance variability, we utilized a single assay kit batch for all analyses. We demonstrated satisfactory within- and between-batch imprecision. Thus, using these two methods, clinically important changes in individual and overall samples might be found.

In our study, although significant differences (shown in Table 1) were observed, some biases were still within an acceptable interval. For example, the results of Fbg, PT/INR, and TT determination were clinically relevant after storage for up to 24 h at 4°C and 25°C; while APTT could be stored for up to 12 h at 4°C and 8 h at 25°C. Of note, the acceptable time interval for APTT determination was longer than recommended in the CLSI H21-A5 guidelines. In contrast, the studies of Wang X et al.^8^ and Wang BL et al.^15^ have shown that the acceptable time intervals for PT and APTT determination are shorter than those recommended in the guidelines. Other studies have suggested that acceptable time intervals for coagulation tests can be extended^13^,^16^,^17^. Kemkes-Matthes et al.^13^ have reported that PT, APTT, Fbg, TT, AT, and D-dimer can be reliably tested after storage for 8 h at room temperature and that the acceptable time interval can easily be extended to 24 h for PT, TT, and D-dimer determination. van Geest-Daalderop et al.^12^ have reported that the acceptable time interval for PT/INR determination is 6 h at 4–6°C, 25°C, and 37°C. Moreover, Oddoze et al.^16^ have reported that the acceptable time interval for APTT determination is 6 h at 4°C and 25°C. In addition, Rao et al.^17^ have reported that plasma and whole blood samples can be tested for PT for up to 24 h and APTT for up to 12 h, when transported either at room temperature or at 4°C.

Although many studies have analyzed the stability of coagulation factor activity in FFP^6^,^19^,^20^,^21^, very few studies have analyzed fresh plasma. In our study, the acceptable time interval for FIX:C determination was 4 h at 4°C and 25°C; while for FVIII:C determination, the only acceptable storage condition was 2 h at 4°C and 25°C. The time interval for FVIII:C determination was shorter, and that for FIX:C determination was consistent, when compared with the recommended times in the CLSI H21-A5 guidelines. The determination of coagulation FVIII:C and FIX:C were used to diagnose congenital or acquired factor deficiency states, distinguish dysproteinemias and protein synthesis disorders, and monitor substitution therapy with FVIII and FIX concentrates in hemophilia A or B^1^,^4^,^6^,^22^,^23^. Furthermore, the determination of FIX was also important for the diagnosis of consumption coagulopathy and hepatic cirrhosis^5^,^22^. Thus, the true activity determination of FVIII and FIX in the patient's plasma was valuable in clinical applications. In Figure 1, we found that FVIII:C and FIX:C were reduced significantly with extended storage time. To avoid FVIII:C and FIX:C becoming lower than the true activity, we carried out a multicenter study, initially reviewed the stability of FVIII:C and FIX:C determination at 25°C and 4°C, and put forward reliable results for clinical diagnosis and treatment. Cardigan et al.^24^ showed that storage of whole blood at room temperature for 8 h resulted in a 23% loss of FVIII:C. Thus, the results of FVIII:C determination at 25°C in our study were similar to those presented by Cardigan et al.^24^, and the results of FIX:C determination were shorter than those presented in that same study^24^.

Our multicenter study has some limitations. First, the study was based on asymptomatic individuals. Second, we used only one type of reagent, instrument, and collection container. Our conclusions can perhaps be corroborated by further study based on different study populations, reagents, instruments, and collection containers.

In conclusion, our results demonstrated that plasma samples tested for Fbg, PT/INR, and TT determination could be safely stored for up to 24 h both at 4°C and 25°C; those tested for APTT measurement could be safely stored for 12 h at 4°C and 8 h at 25°C; those tested for FIX:C measurement could be safely stored for 4 h at 4°C and 25°C; and those tested for FVIII:C could be safely stored for only 2 h at 4°C and 25°C, suggesting that FVIII:C should be immediately measured within 2 h after collection in our laboratory. Thus, clinical samples should only be stored for these time frames prior to testing.

## Methods

### Participating centers

The study was carried out in two laboratories, including the First Affiliated Hospital of Zhejiang University (Center 1) and The First People's Hospital of Hangzhou (Center 2). Both Centers, 1 and 2, used the same blood collection system (Becton Dickinson, Franklin Lakes, USA), Sysmex CA7000 system (Sysmex, Kobe, Japan), Siemens reagents (Siemens, Marburg, Germany), and International Sensitivity Index (ISI, ISI = 0.99).

### Sample size

Sample size estimates were calculated using a post hoc power of analysis with two size effects (0.8/0.9) and alpha levels (0.05/0.1). A sample size of 72 patients (36 per group) was appropriate.

### Patients

The study involved 36 asymptomatic individuals who visited the First Affiliated Hospital of Zhejiang University (Center 1) and 36 asymptomatic individuals who visited the First People's Hospital of Hangzhou (Center 2) for physical examination in September 2013. The patients of Center 1 included 18 men and 18 women, with a median age of 44 years old (range, 19–75 years old); the patients of Center 2 included 18 men and 18 women, with a median age of 45 years old (range, 21–78 years old).

### Ethics statement

This study was approved by the Ethics Committees of the First Affiliated Hospital of Zhejiang University and The First People's Hospital of Hangzhou, China. Patients provided written informed consent for their samples to be used in the study.

### Assays

Venipunctures were performed in the morning following a 12-h fast. From each patient, a 5.4-ml venous whole blood sample was collected into a tube containing 0.109 M sodium citrate as an anticoagulant (Becton Dickinson, Franklin Lakes, USA) at a blood to anticoagulant ratio of 9:1. The 72 samples were centrifuged (10 min, 3000 *g*) to obtain fresh plasma without platelets and cells, and each sample was divided into 13 Eppendorf tubes and capped. The Eppendorf tubes used for the aliquots were composed of a nonactivating plastic. One split tube was tested immediately, which served as the baseline (0 h) results, while the remaining 12 split tubes were tested, respectively, after storage for 2, 4, 6, 8, 12, and 24 h at 25°C and 4°C.

In Centers 1 and 2, the plasma samples were tested by a coagulation method for APTT, PT/INR, TT, Fbg, FVIII:C, and FIX:C using a Sysmex CA7000 system (Sysmex, Kobe, Japan) and Siemens reagents (Siemens, Marburg, Germany): Dade Actin activated cephaloplastin reagent (lot 547713), Dade Thrombin reagent (lot 538065), Thromborel S (lot 545515), Test Thrombin reagent (lot 42780), Coagulation factor VIII-deficient plasma (lot 546576), and Coagulation factor IX-deficient plasma (lot 500870B), respectively. In addition, INR was transformed by PT according to the formula: INR = (PT/mean normal prothrombin time)^ISI^. The compositions of Siemens reagents were liquid rabbit brain cephalin with plasma activator, lyophilized bovine thrombin, lyophilized human placental thromboplastin, lyophilized human bovine thrombin and bovine albumin, and lyophilized human plasma with a residual FVIII:C and FIX:C of ≤1% for use in the determination of APTT, Fbg, PT, TT, FVIII:C, and FIX:C. The results were expressed in s (PT, APTT, and TT), g/l (Fbg level), and % (FVIII:C and FIX:C).

Two mixed pools having normal and abnormal target results were prepared from freshly collected plasma and were used to measure APTT, Fbg, PT, TT, FVIII:C, and FIX:C 20 times in 2 h to assess within-batch imprecision. Commercial quality control products (SIEMENS) with target results in normal/abnormal ranges were used to measure APTT, Fbg, PT, TT, FVIII:C, and FIX:C on 20 separate days to calculate between-batch imprecision. The manufacturer's product information for the assays claims that between- and within-batch imprecision should be <15% and <5%, respectively, for PT, APTT, Fbg, and TT; between-batch imprecision should be <10% for FVIII:C and FIX:C; and within-batch imprecision should be <10% (normal) and <3% (abnormal) for FVIII:C and FIX:C, respectively.

### Statistical analyses

The coagulation test and factor activity results were reported as mean ± standard deviation. The results following storage for 2, 4, 6, 8, 12, and 24 h at 25°C and 4°C were compared with the baseline results by using paired t-tests. To assess the stability of the coagulation tests and factor activities, the percentage changes compared to the baseline results were calculated [(result at storage time X – result at baseline)/result at baseline] and averaged for each time point^2^,^12^,^13^,^14^. According to the study by van Geest-Daalderop et al., a clinically relevant difference was defined as a mean percentage change of greater than 10%. If the number of individuals with a greater than 10% percentage change was less than 25% of the total sample number (i.e., 18 in our study), the effect of the given pre-analytical variable was termed moderate, whereas if more than 25% of the samples had a greater than 10% change, the effect was deemed large^12^. P values < 0.05 were considered statistically significant. Statistical analyses were performed using SPSS software, version 16.

## Author Contributions

Z.X.S. designed the experiments. L.M.F., Y.Z. and H.C.Z. performed the experiments. L.M.F. and Y.Z. wrote the main manuscript text. All authors reviewed the manuscript.

## Figures and Tables

**Table 1:** The results of PT/INR, APTT, Fbg, TT, FVIII:C, and FIX:C measurements

	Baseline	Temperature	2 h	4 h	6 h	8 h	12 h	24 h
PT (s)	11.9 ± 2.7	4°C	11.6 ± 2.6*	11.6 ± 2.5*	11.7 ± 2.5*	11.7 ± 2.5*	11.9 ± 2.5*	12.7 ± 2.9*
		25°C	11.5 ± 2.6*	11.4 ± 2.5*	11.4 ± 2.4*	11.3 ± 2.4*	11.4 ± 2.3*	12.8 ± 3.0*
INR	1.08 ± 0.25	4°C	1.05 ± 0.23*	1.05 ± 0.23*	1.06 ± 0.22*	1.06 ± 0.23*	1.08 ± 0.23*	1.15 ± 0.26*
		25°C	1.05 ± 0.23*	1.04 ± 0.22*	1.03 ± 0.21*	1.03 ± 0.21*	1.04 ± 0.21*	1.16 ± 0.27*
APTT (s)	28.3 ± 5.8	4°C	28.4 ± 6.0	29.1 ± 6.3*	29.7 ± 6.6*	29.7 ± 6.2*	30.7 ± 6.7*	33.4 ± 7.9*
		25°C	28.2 ± 5.9	28.8 ± 6.1*	29.5 ± 6.3*	30.2 ± 6.4*	32.0 ± 6.7*	34.2 ± 7.6*
TT (s)	17.4 ± 0.79	4°C	17.3 ± 0.8*	17.2 ± 0.8*	17.2 ± 0.8*	17.1 ± 0.8*	17.2 ± 0.8*	17.5 ± 0.8*
		25°C	17.4 ± 0.8*	17.3 ± 0.8*	17.2 ± 0.8*	17.3 ± 0.8*	17.2 ± 0.8*	17.8 ± 0.9*
Fbg (g/L)	2.74 ± 0.81	4°C	2.79 ± 0.85*	2.82 ± 0.85*	2.79 ± 0.83*	2.82 ± 0.84*	2.79 ± 0.84*	2.81 ± 0.83*
		25°C	2.76 ± 0.83*	2.78 ± 0.83*	2.77 ± 0.83*	2.79 ± 0.83*	2.78 ± 0.85*	2.77 ± 0.82
FVIII:C (%)	141.8 ± 42.2	4°C	132.1 ± 40.0*	121.1 ± 36.9*	112.6 ± 35.3*	109.6 ± 37.1*	101.2 ± 33.8*	79.4 ± 25.7*
		25°C	133.4 ± 41.0*	121.1 ± 37.0*	108.5 ± 34.1*	98.9 ± 33.3*	83.5 ± 28.7*	72.4 ± 22.9*
FIX:C (%)	101.1 ± 22.7	4°C	97.4 ± 21.2	94.4 ± 20.5*	91.8 ± 19.4*	91.9 ± 20.4*	89.1 ± 20.0*	87.4 ± 17.8*
		25°C	98.0 ± 22.0*	94.3 ± 20.4*	91.1 ± 19.5*	89.6 ± 19.2*	85.2 ± 17.7*	84.8 ± 16.6*

*, p < 0.05 compared with baseline results.

Abbreviations: APTT, activated partial thromboplastin time; Fbg, fibrinogen; PT, prothrombin time; INR, the international normalized ratio; TT, thrombin time; FVIII:C, factor VIII activity; and FIX:C, factor IX activity.

**Table 2:** The stabilities of PT/INR, APTT, Fbg, TT, FVIII:C, and FIX:C

	Mean percentage change (%)													
	2 h		4 h		6 h		8 h		12 h		24 h		Acceptable Time (h)	
	4°C	25°C	4°C	25°C	4°C	25°C	4°C	25°C	4°C	25°C	4°C	25°C	4°C	25°C
PT (s)	−2.66	−3.29	−2.69	−4.20	−2.08	−4.62	−2.06	−4.90	−0.37	−3.80	6.58	5.97	24	24
INR	−2.63	−3.26	−2.66	−4.16	−2.06	−4.58	−2.04	−4.85	−0.36	−3.65	6.51	6.81	24	24
APTT (s)	0.13	−0.43	2.71	1.48	4.59	3.98	4.82	6.56	8.01	12.92*	17.46*	20.4*	12	8
TT (s)	−0.51	−0.26	−1.23	−0.85	−1.11	−0.93	−1.84	−0.79	−1.29	−1.25	0.31	2.09	24	24
Fbg (g/L)	1.43	0.67	2.56	1.24	1.85	0.86	2.56	1.71	1.68	1.25	2.34	0.86	24	24
FVIII:C (%)	−6.98	−6.09	−14.72*	−14.63*	−20.82*	−23.71*	−23.05*	−30.72*	−29.13*	−41.62*	−44.30*	−49.13*	2	2
FIX:C (%)	−3.58	−3.07	−6.43	−6.58	−8.91*	−9.67*	−8.76*	−11.08*	−11.63*	−15.32*	−13.17*	−15.59*	4	4

*indicates that changes >10% in individual samples occurred in >25% of samples.

**Table 3:** The optimal storage conditions of coagulation tests and factors in fresh plasma

Reference	Temperature	PT	INR	APTT	Fbg	TT	FVIII:C	FIX:C
Zhao et al.^2^	4°C	24 h	-	8 h	24 h	24 h	-	-
	25°C	24 h	-	8 h	24 h	24 h	-	-
Wang et al.^8^	4°C	6 h	-	6 h	-	-	-	-
	25°C	4 h	-	4 h	-	-	-	-
Saghir et al.^10^	4°C	-	-	2 h	-	-	-	-
	25°C	4 h	-	2 h	-	-	-	-
van Geest-Daalderop et al.^12^	4°C	6 h	6 h	-	-	-	-	-
	25°C	6 h	6 h	-	-	-	-	-
Kemkes-Matthes et al.^13^	4°C	-	-	-	-	-	-	-
	25°C	24 h	-	8 h	8 h	24 h	-	-
Wang et al.^15^	4°C	-	-	-	-	-	-	-
	25°C	8 h	-	6 h	8 h	8 h	-	-
Oddoze et al.^16^	4°C	-	-	6 h	-	-	-	-
	25°C	-	-	6 h	-	-	-	-
Rao et al.^17^	4°C	24 h	-	12 h	-	-	-	-
	25°C	24 h	-	12 h	-	-	-	-

-: no result.

## References

[b1] Lippi G., Guidi G. C., Mattiuzzi C. & Plebani M. Preanalytical variability: the dark side of the moon in laboratory testing. Clin Chem Lab Med 44, 358–365 (2006). 16599826 10.1515/CCLM.2006.073

[b2] Zhao Y. & Lv G. Influence of temperature and storage duration on measurement of activated partial thromboplastin time, D-dimers, fibrinogen, prothrombin time and thrombin time, in citrate-anticoagulated whole blood specimens. Int J Lab Hematol 35, 566–570 (2013). 23718185 10.1111/ijlh.12113

[b3] Loeliger E. A., van den Besselaar A. M. & Lewis S. M. Reliability and clinical impact of the normalization of the prothrombin times in oral anticoagulant control. Thromb Haemost 53, 148–154 (1985). 3992514

[b4] Mulder R. *et al.* Associations between high factor VIII and low free protein S levels with traditional arterial thrombotic risk factors and their risk on arterial thrombosis: results from a retrospective family cohort study. Thromb Res 126, e249–254 (2010). 20705334 10.1016/j.thromres.2010.07.013

[b5] Prelipcean C. C. *et al.* [Liver cirrhosis--procoagulant stasis]. Rev Med Chir Soc Med Nat Iasi 115, 678–685 (2011). 22046771

[b6] Omidkhoda A. *et al.* A comparative study of the effects of temperature, time and factor VIII assay type on factor VIII activity in cryoprecipitate in Iran. Blood Transfus 9, 394–399 (2011). 21839017 10.2450/2011.0064-10 PMC3200408

[b7] Cong, Y. L. & Wang, S. H. J. editor. Jinri linchuang jianyanxue: Beijing: Military medical publisher. 204–207p (1997).

[b8] Wang X., Ma J. Z. H. & Hao Z. L. Influence of storage time of PT and APTT at different temperature. Journal of Hebei Medical University 23, 108–109 (2002).

[b9] Adcock D. M. *et al.* Collection, transport, and processing of blood samples for testing plasma-based coagulation assays and molecular hemostasis assays. Approved guideline: fifth edition (H21-A5). Clinical and Laboratory Standards Institute 28, 1–33 (2008).

[b10] Mohammed Saghir S. A., Al-Hassan F. M., Alsalahi O. S., Abdul Manaf F. S. & Baqir H. S. Optimization of the storage conditions for coagulation screening tests. J Coll Physicians Surg Pak 22, 294–297 (2012). 22538033

[b11] Adcock D., Kressin D. & Marlar R. A. The effect of time and temperature variables on routine coagulation tests. Blood Coagul Fibrinolysis 9, 463–470 (1998). 9818995 10.1097/00001721-199809000-00002

[b12] van Geest-Daalderop J. H., Mulder A. B., Boonman-de Winter L. J., Hoekstra M. M. & van den Besselaar A. M. Preanalytical variables and off-site blood collection: influences on the results of the prothrombin time/international normalized ratio test and implications for monitoring of oral anticoagulant therapy. Clin Chem 51, 561–568 (2005). 15650035 10.1373/clinchem.2004.043174

[b13] Kemkes-Matthes B., Fischer R. & Peetz D. Influence of 8 and 24-h storage of whole blood at ambient temperature on prothrombin time, activated partial thromboplastin time, fibrinogen, thrombin time, antithrombin and D-dimer. Blood Coagul Fibrinolysis 22, 215–220 (2011). 21297453 10.1097/MBC.0b013e328343f8bf

[b14] Salvagno G. L. *et al.* Influence of temperature and time before centrifugation of specimens for routine coagulation testing. Int J Lab Hematol 31, 462–467 (2009). 18371059 10.1111/j.1751-553X.2008.01058.x

[b15] Wang B. L., Guo W. L. & Pan B. S. H. Influence of storage time at room temperature on routine coagulation tests. Chinese Journal of laboratory medicine 34, 595–597 (2011).

[b16] Oddoze C., Lombard E. & Portugal H. Stability study of 81 analytes in human whole blood, in serum and in plasma. Clin Biochem 45, 464–469 (2012). 22285385 10.1016/j.clinbiochem.2012.01.012

[b17] Rao L. V., Okorodudu A. O., Petersen J. R. & Elghetany M. T. Stability of prothrombin time and activated partial thromboplastin time tests under different storage conditions. Clin Chim Acta 300, 13–21 (2000). 10958859 10.1016/s0009-8981(00)00288-6

[b18] Zürcher M., Sulzer I., Barizzi G., Lammle B. & Alberio L. Stability of coagulation assays performed in plasma from citrated whole blood transported at ambient temperature. Thromb Haemost 99, 416–26 (2008). 18278194 10.1160/TH07-07-0448

[b19] Alhumaidan H., Cheves T., Holme S. & Sweeney J. Stability of coagulation factors in plasma prepared after a 24-hour room temperature hold. Transfusion 50, 1934–1942 (2010). 20412526 10.1111/j.1537-2995.2010.02648.x

[b20] von Heymann C. *et al.* Thawing procedures and the time course of clotting factor activity in fresh-frozen plasma: a controlled laboratory investigation. Anesth Analg 103, 969–974 (2006). 17000814 10.1213/01.ANE.0000240416.56803.5B

[b21] Scott E., Puca K., Heraly J., Gottschall J. & Friedman K. Evaluation and comparison of coagulation factor activity in fresh-frozen plasma and 24-hour plasma at thaw and after 120 hours of 1 to 6 degrees C storage. Transfusion 49, 1584–1591 (2009). 19413730 10.1111/j.1537-2995.2009.02198.x

[b22] Ingerslev J. Efficacy and safety of recombinant factor VIIa in the prophylaxis of bleeding in various surgical procedures in hemophilic patients with factor VIII and factor IX inhibitors. Semin Thromb Hemost 26, 425–432 (2000). 11092219 10.1055/s-2000-8463

[b23] Bassus S. *et al.* Platelet-dependent coagulation assays for factor VIII efficacy measurement after substitution therapy in patients with haemophilia A. Platelets 17, 378–384 (2006). 16973498 10.1080/09537100600757448

[b24] Cardigan R. *et al.* Coagulation factor content of plasma produced from whole blood stored for 24 hours at ambient temperature: results from an international multicenter BEST Collaborative study. Transfusion 51 Suppl 1, 50S–57S (2011). 21223296 10.1111/j.1537-2995.2010.02963.x

